# Annotations

**Published:** 1890-09-20

**Authors:** 


### Annotations.

**Duelling and Development.**

M. Jules Simon blushes at the recent outbreak of duelling among his ridiculous fellow-countrymen. There is nothing your true Frenchman hates more than to be made to appear ridiculous; and his very dread of such a possibility is constantly placing him in absurd situations. Duelling could not possibly survive in a nation endowed with good human brains. The French inherit too many of the trickish and mimicking aptitudes of the smaller monkeys, and too little of the solid and capable intelligence of the higher orders of the primates. The Englishman has long ago given up duelling, on the same ground that his remote ancestors gave up the "ordeal by poison," namely, that it never did and never could establish the rights and wrongs of a question. Even a Frenchman has brains enough to see that the duel can no more establish the rights and wrongs of a question than the ordeal by poison. The conclusion therefore is inevitable that, though the Frenchman may be clever and smart at tricks and mimicry, and all sorts of things that may be learned from others, yet in solid, broad, original, and judicial intellect, he is markedly deficient. The Englishman, looking on in imagination at a French duel, feels conscious of his superiority in everything that constitutes true intellectual capacity. He laughs to himself at the attitude of the two jackanapes, who are behaving like play actors or wooden marionettes in real earnest. If he were to follow the promptings of his instinct he would do to these two men what he would to a couple of strange terriers, which, meeting each other for the first time, should fall to fighting because they had no more sense. He would, if such a thing were handy, turn a garden hose upon them to cool their ridiculous excitement; or, failing that, would get a few buckets of water at the nearest pump and souse them well with it, until each of the half-drowned mountebanks should look as ridiculous to the other as they both did to him when they were trying to prod one another with swords. Let no French reader be hurt by this plain kind of speech. We feel that the French are our very good neighbours, and that they have many excellent qualities which make us love them. Being thus our neighbours and dear friends we admit a kind of responsibility for them, and feel honestly ashamed that they should behave themselves so absurdly on the village green of the civilised world. For our sakes, if not for their own, they should make an effort to get beyond the Marmoset stage of development.

**What the Vegetarians Want to Know.**

"What the vegetarians want is to know what the right and proper foods are." That seems to be the net result of the Vegetarian Congress held at the Memorial Hall last week. Now there are a good many persons among the vegetarians who are cultured, clever, and reasonable. Is it really the fact, as Mr. Manning, who appears to live on nuts, says it is, that this is their attitude of mind? If it be so, we would ask, with a kind of plaintive despair, where they expect to find the oracle which will tell them exactly what to eat. It must be plain that their problem will have to be solved either by all men, or by some selected men, or by each man for himself. We know of no other method of settling any kind of human business. But all men are out of the question; for it has never yet happened in the history of the world that all have agreed on any single point. Must, then, the appeal be made to a limited number of selected individuals? And if so, who are to be the favoured few? Are they to be doctors, or lawyers, or Cambridge professors, or dark skinned persons who have a taste for human steak, or young ladies who live on real tea, and imaginary bread and butter? The vegetarians certainly will not select doctors for their prophets; for it is to be feared that there are considerable numbers of medical Elijahs who regard vegetarians as "Children of Baal"; and the original Elijah had a "short way with dissenters" of the Baalite type. Failing the doctors, there really seems to be no class of men to whom appeal can be made. The "City Fathers" are scarcely available, at any rate from a purely vegetarian point of view; and the clergy, though some of them, perhaps, are involuntary vegetarians, seem to be like Ephraim, "joined to their idols" of good living, and had better be "let alone." As for lawyers, one never quite knows what a lawyer eats, though it is pretty certain that he does not dine off parchment. The only possibility left seems to be that every man must be "fully persuaded in his own mind," and find out by personal experience what kind of diet is best for him. He will get a certain amount of help from the customs of the mass of mankind, and a little more from common sense doctors. Armed with these weapons he will probably manage to lead a fairly comfortable and healthy life, whether he inclines to flesh, or to vegetables, or even pins his faith to an exclusive diet of nuts.

**Kent Christians.**

There seems no just ground for regarding the people of Kent as other than Christians. And yet if we are to take it for granted that the resident population of the Garden of England are average representatives of the Christian faith we have some reason for calling a mental halt, and giving a little thought to one or two questions. All of us were not born in Kent; and therefore we cannot all be expected to see its peculiar institutions exactly as the native sees them. The present writer, for example, happens to be a Yorkshireman; and he is very familiar with the ways of that county. Some of its bad characteristics may not strike him as they strike a stranger. If that be so he is sorry for it, and he hereby requests every stranger who may visit Yorkshire and notice any gross brutalities among the people to proclaim them upon the house tops to as many people as can be prevailed upon to hear. In like manner he asks for the liberty of calling attention to one particular thing which has struck him during a summer sojourn in the "hop country." Paddock Wood is not far from the centre of the chief hop growing district in Kent. Driving through the lanes near Paddock Wood the other evening the writer came upon a kind of building such as he had never seen in any other part of England or Scotland. For the moment he could not imagine what the use of it might be. It was a very long, low, brick structure, having its whole length occupied by doors, which seemed like cottage doors. There was not a window to be seen of any sort. The building was only a single story high and appeared to have doors along its entire length on both sides. Some of the doors were open, and several interior compartments were thereby rendered visible. They were all of exactly the same size and shape; that is to say, about seven or eight feet long, five feet wide, and nine or ten feet high. What were they for, the stranger will ask? They were "hoppers'" houses. Each compartment was a house in itself, and served for dining-room, drawing-room, library, kitchen, scullery, bedroom, bath-room, and every other purpose whatsoever. There was not a chair to be seen, nor a table, nor a bed, nor a fire-place, nor, as we have said, even a window. There were fires lighted in one or two of the compartments, but they were on the floor. There was no convenient hole in the roof for the smoke to escape from as there is in the hut of a savage, or of prosperous Irish tenants. The door was flung wide open, and through it the smoke escaped. Where do the people sleep? was asked of the driver. On the floor. How many sleep in that one compartment? The whole of one family; sometimes it may consist simply of a man and his wife; and at other times of the man and his wife and six or seven children. But how can they breathe in such a place? They leave the door open all night. But where do the unmarried boys and girls, and young men and young women sleep? Where they can and where they like. And there are tens of thousands of these people here for five or six weeks? Yes! It is to be presumed that these hoppers are all baptised Christians; that their employers are baptised Christians too; and that the clergy, who are somewhat numerous under the shadow of Canterbury Cathedral, and the doctors, and the rich residents from London are also baptised Christians. Concerning this there are two things whichmay be said. If a Mahommedan or other non-Christian person were to see such things he would think Christianity a religion for pigs. That is one thing. The other is that if the writer did not know anything of Christianity, except by such fruits as these, he would be an atheist rather than a Christian. We are a terrible set of "humbugs," fellow Christians!

